# Body Mass Index and Hemoglobin A1c Correlate with Clinical Needs After COVID-19 Vaccination in the Veterans Affairs System

**DOI:** 10.3390/jcm14238271

**Published:** 2025-11-21

**Authors:** Jay Pendse, Gabriela Jordan, Binhuan Wang, Craig Tenner, Brenda Dorcely, Robert J. Ulrich, Kevin Zhang, Sabrina Felson, Melanie Jay, José O. Alemán

**Affiliations:** 1 Holman Division of Endocrinology, Department of Medicine, NYU Grossman School of Medicine, New York, NY 10016, USA; 2 Laboratory of Translational Obesity Research, New York, NY 10016, USA; 3 Margaret Cochran Corbin (Manhattan) Campus, VA New York Harbor Healthcare System, New York, NY 10010, USA; 4 Department of Medicine, Division of General Internal Medicine and Clinical Innovation, NYU Grossman School of Medicine, New York, NY 10016, USA; 5 Department of Population Health, Division of Epidemiology, NYU Grossman School of Medicine, New York, NY 10016, USA; 6 Departments of Medicine and Population Health, NYU Grossman School of Medicine, New York, NY 10016, USA; 7 Division of Infectious Diseases and Immunology, Department of Medicine, NYU Grossman School of Medicine, New York, NY 10016, USA

**Keywords:** obesity, diabetes, COVID-19, veterans

## Abstract

**Background:** Throughout the course of the COVID-19 pandemic, clinicians recognized that individuals with metabolic syndrome, including elevated body mass index (BMI) and type 2 diabetes, have increased clinical care requirements and worsened outcomes during COVID-19 infection. With the availability of COVID-19 vaccines, it was unknown whether vaccination could mitigate the clinical outcomes among patients with metabolic syndrome. In this study, we sought to determine whether BMI and hemoglobin A1c are associated with a risk of breakthrough infection and increased clinical needs among patients who have been fully vaccinated against COVID-19. **Methods**: We conducted a retrospective cohort study of patients in the Veterans Affairs healthcare system who were vaccinated against COVID-19 between 1 December 2020 and 22 August 2021. We sampled a random subset of 549,344 patients from a total of over 1 million de-identified patients greater than age 18 who were vaccinated between 1 December 2020 and 22 August 2021, without a prior positive COVID-19 test in the VA healthcare system data warehouse. The primary study outcomes were breakthrough COVID-19 infections after vaccination and hospitalization due to breakthrough COVID-19 infections. **Results:** We identified 480,129 patients with available BMI and hemoglobin A1c data; of these, all had data available for the covariates of race, ethnicity, sex, and age, and 467,283 had data available for district as well. Adjusting for those covariates, Cox proportional hazards modeling for time from vaccination until breakthrough infection demonstrated that higher BMI (HR per unit 1.015, *p* < 0.001) and hemoglobin A1c were associated with an increased risk of infection (HR per unit 1.063, *p* < 0.001). The number of patients from this set who developed breakthrough infections within the study period was 8903 (9146 if those with missing district data were included). The average age of fully vaccinated patients with breakthrough COVID-19 infection within six months of full vaccination was 64.5. The average BMI was 31.2 ± 6.2 and the average A1c was 6.34 ± 1.5. Adjusting for the above covariates, multivariable logistic regression trends towards significance, with an increased risk of hospitalization due to breakthrough COVID-19 infection with increased BMI (HR per unit 1.010, *p* = 0.052), and was statistically significant for increased hemoglobin A1c (HR per unit 1.150, *p* < 0.010). **Conclusions:** This study identifies BMI and hemoglobin A1c as risk factors for breakthrough COVID-19 infection among fully vaccinated patients in the US veteran population.

## 1. Introduction

Early in the COVID-19 pandemic, it was noted that the patients who were younger and otherwise healthy who were requiring a higher level of care were often overweight or obese. Type 2 diabetes was also identified as a risk factor for severe infection and poor outcomes early in the pandemic. Since then, numerous studies have demonstrated an association between elevated body mass index (BMI) and Type 2 diabetes and poor outcomes in COVID-19 infections, including higher rates of mortality, intubation, and intensive care unit admission.

One study conducted at New York University in 2020 found that in patients diagnosed with COVID-19, a BMI greater than 40 was among the strongest risk factors for critical illness, including requiring intensive care, ventilation, discharge to hospice, or death [1]. Another nation-wide study found that overweight and obesity were risk factors for requiring invasive mechanical ventilation, and obesity was a risk factor for hospitalization and death, particularly among adults age < 65 years [2]. A single-center study in New Jersey found that the mean BMI of patients requiring intubation was significantly higher than that of non-intubated patients [3]. In Germany, a retrospective review of 184 patients with acute respiratory distress syndrome due to COVID-19 found that extreme obesity (BMI > 40 kg/m^2^) was the strongest predictor for in-hospital mortality [4].

There are multiple proposed mechanisms for this relationship between obesity and poor outcomes in COVID-19 infections. Regarding obesity, many studies have shown an overactive inflammatory and immune response, with elevated inflammatory markers such as IL-6 and CRP [5], both of which were found to be associated with worse outcomes in COVID-19 infections. A different possible mechanism is through increased thromboembolic risk, which is known to be higher in patients with obesity and thromboembolism, such as pulmonary embolism and deep vein thrombosis [6], which are well-established complications of COVID-19 infections. Body habitus with obesity can also cause increased abdominal pressure and limited chest wall expansion, which can lead to insufficient respiratory compensation in acute hypoxic respiratory failure [7]. Another proposed mechanism involves the ACE-2 receptor. Early in the pandemic, it was discovered that the SARS-CoV-2 virus spike protein S binds to ACE-2 receptors in lung tissue, which would allow for cell entry and subsequent pulmonary infection.

Studies have also found increased expression of ACE-2 receptors in adipose tissue, which avidly binds the SARS-CoV-2 virus protein and allows for greater tissue invasion, perhaps even more than in lung tissue. This suggests that patients with obesity have greater adipose tissue and therefore present a greater opportunity for the virus to enter the body [8].

Numerous studies have also demonstrated an association between diabetes and inpatient hyperglycemia and worse outcomes. A 2020 study of over 10,000 US veterans diagnosed with COVID-19 found that diabetes was associated with a higher risk of requiring mechanical ventilation [9]. Similarly, a multicenter U.S. study assessed inpatient poorly controlled hyperglycemia and found that COVID-19 patients with diabetes and/or uncontrolled hyperglycemia had a longer length of stay and higher mortality than patients without diabetes or uncontrolled hyperglycemia [10]. A study of over 70,000 Swedish subjects found that at 6 months following a COVID-19 infection, there was an excess burden of diabetes, obesity, and elevated hemoglobin A1c (HbA1c) [11].

Some of the proposed mechanisms for the relationship between diabetes and poor COVID-19 outcomes overlap with those for obesity. Diabetes is also associated with a pro-inflammatory state and has been shown to result in increased inflammatory markers, such as acute phase reactants, cytokines, and chemokines. Since COVID-19 infections are linked to significant inflammation and the release of cytokines, it is thought that patients with diabetes who are infected with COVID-19 are more likely to develop a more severe inflammatory response. One retrospective study found a positive correlation between HbA1c and higher ferritin, CRP, fibrinogen, and ESR [12], all of which were associated with worse outcomes in COVID-19. Another proposed mechanism is through the impaired immune response among patients with diabetes, which overall increases the risk of more severe infections.

Vaccines against COVID-19 have become widely available and have been shown to be highly effective in preventing severe disease and mortality in vaccinated patients exposed to the virus. However, breakthrough infections have occurred, and we are now learning which patients are at a higher risk of breakthrough infection and, among those, which are at a higher risk for severe infection and worse outcomes. Other studies have shown that co-morbidities, such as overweight and diabetes, were frequently seen in patients with severe illness in breakthrough COVID-19 infections [13]. Knowing that elevated BMI and poor glycemic control are associated with worse outcomes prior to vaccination, we sought to evaluate whether these factors are associated with the risk of breakthrough infection and increased clinical needs among patients who have been fully vaccinated against COVID-19.

## 2. Materials and Methods

Our study was conducted in the Veterans Health Administration healthcare system, the healthcare system of the U.S. Department of Veterans Affairs (VA), which includes over nine million patients across the United States. Early in the COVID-19 pandemic, the VA system created a National VA COVID-19 shared data resource, which allowed VA investigators across the country to easily access a repository of COVID-19 data. We sought IRB approval for the use of the National VA COVID-19 shared data resource when available in August 2020. This resource accumulated deidentified live data during the COVID-19 pandemic for VA research use, and IRB approval was a requisite to access this non-human subjects data. Using this database, we retrospectively evaluated deidentified records of patients who were fully vaccinated against COVID-19 and developed breakthrough infections between 1 December 2020 and 3 October 2021, and explored the role of elevated BMI and HbA1c in the risk of breakthrough infections and having increased clinical needs, such as requiring hospitalization. The rationale for the limited follow-up of the study is that we aimed to capture a similar time period to the COVID-19 pandemic, when there were no vaccinations available between February and December 2020, in a natural experiment framework.

We used the VA’s Corporate Data Warehouse via the VA Informatics and Computing Infrastructure (VINCI). As there are several million patients in the database, we took a randomly sampled subset of about 60% of those patients in order to keep the remainder of patients for use in testing our models in the future. Among those, we identified nearly 549,344 patients older than age eighteen who were vaccinated between 1 December 2020 and 22 August 2021 without a positive COVID-19 test within six weeks of receiving their first vaccine. We included patients who developed breakthrough infections within six months of full vaccination. Among those, 480,129 patients had available BMI and HbA1c data; of these, all had data available for the covariates of race, ethnicity, sex, and age, and 467,283 had data available for district as well. We identified 9146 patients who had a breakthrough infection during the study period. We selected hemoglobin A1c as a marker of diabetes instead of a diagnosis of diabetes in order to be able to assess glycemic status as a continuous variable. We similarly chose BMI as a marker of overweight and obesity in order to assess weight status on a continuum. We sampled HbA1c and BMI measurements using the most recent value on record available up to January 2018, in particular to capture less frequent HbA1c measurements. BMI and A1c values are a single time point for the most recently available value. Using Cox proportional hazards and Kaplan–Meier curves, we compared the rate of breakthrough infection over time among patients of various HbA1c and BMI ranges. Using multivariable logistic regression, we assessed the role of BMI and HbA1c on the rate of hospitalization.

## 3. Results

**Figure 1:**
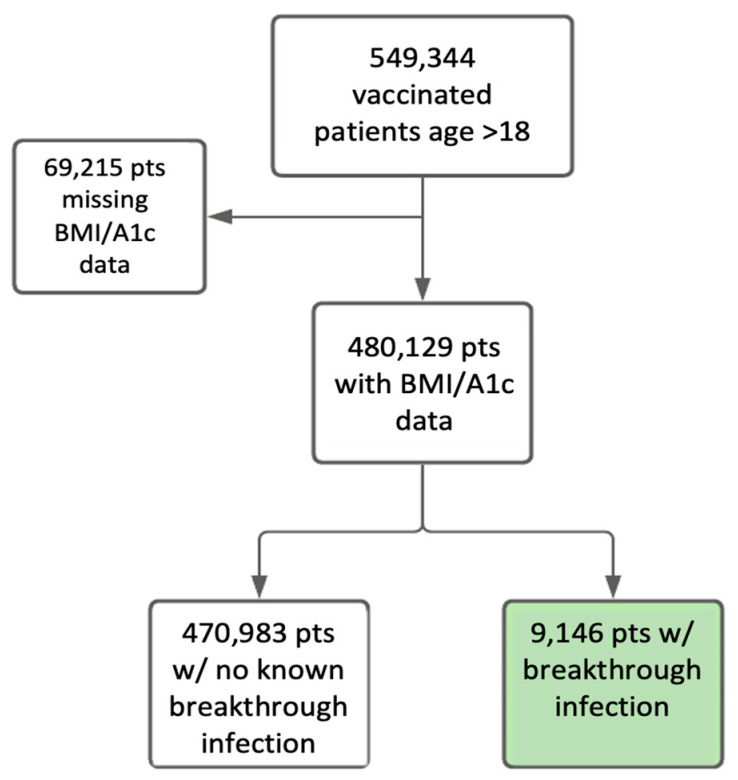
TREND diagram of virtual cohort study. (BMI: Body Mass Index, A1c: Hemoglobin A1c).

The flow diagram of participants is presented in Figure 1, leading to the selection of 9146 adult patients vaccinated within the defined time period and with available BMI and HbA1c data (8903 with complete data). Table 1 shows the subset of 549,344 patients who were vaccinated in the VA system. The patients in the “breakthrough infection” category include patients who had a breakthrough infection within six months of complete vaccination, identified by a positive PCR test. The vaccines used in these subjects included Pfizer *n* = 254,037; Moderna *n* = 259,448; and Janssen *n* = 35,859. Only 0.75% of the total vaccinated population had breakthrough infections in this time period. The category of “no breakthrough infection” means that they had not had a documented infection after six months since vaccination. The final category includes patients who were vaccinated within the last six months, and we did not yet have a complete set of breakthrough infection data at that time. Given the nature of the VA population, the majority of patients are older, white, non-Hispanic males, which is reflective of the general VA population. The patients are also well distributed across the different regions of the country. The average age of fully vaccinated patients with breakthrough COVID-19 infection within six months of full vaccination is 64.5 ± 14.3. The average BMI is 31.2 ± 6.2 kg/m^2^ and the average A1c was 6.34 ± 1.5% (see Table 2 and Table 3). It is worth noting that the total number of breakthrough infections here is limited to those within six months of vaccination as a way to clearly categorize the patient groups. The 9146 patients included in the flowchart of patients included in the data analysis include some of those who have had breakthroughs but were not yet six months out from their vaccination.

The median and mean HbA1c were slightly higher among the patients with breakthrough infections (6.34% vs. 6.2%). Using a similar unpaired *t*-test, we determined that the *p*-value for the difference between mean HbA1cs was statistically significant (*p* < 0.001).

**Figure 2:**
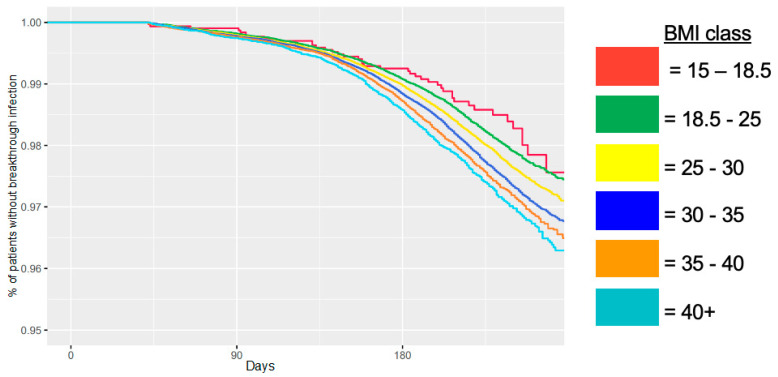
BMI and rate of breakthrough infection over time since second COVID-19 vaccination dose.

Using Cox proportional hazards modeling, we show that HbA1c is a greater hazard for breakthrough infection than BMI. The Cox tables show coefficients and *p*-values for Cox proportional hazards modeling for time from vaccination until breakthrough infection, adjusting for covariates, including race, ethnicity, sex, age, and district (Table 4). We used models that included BMI, HbA1c, both, or neither. We found that hazard ratios for breakthrough infection are impacted by BMI, HbA1c, and both. In the model that included BMI without HbA1c, the hazard ratio (HR) for risk of breakthrough infection was 1.017 (*p* < 0.001). In the model with HbA1c without BMI, the HR was 1.077 (*p* < 0.001). When both BMI and HbA1c were added to the model, the HRs were 1.015 (*p* < 0.001) and 1.063 (*p* < 0.001), respectively. Both covariates are highly significant; however, HbA1c portends a higher risk. In other words, a one-point increase in HbA1c correlates with a higher risk of breakthrough infection than adding one point to BMI. Using a Kaplan–Meier curve, we graphically show that patients with the highest BMI class had the highest rates of breakthrough infection at a given time after full vaccination (Figure 2). It is noteworthy that the y-axis is only 5%, in order to highlight the differences. The patients in the lowest BMI category appear to have a lower risk than patients within the normal weight category.

**Figure 3:**
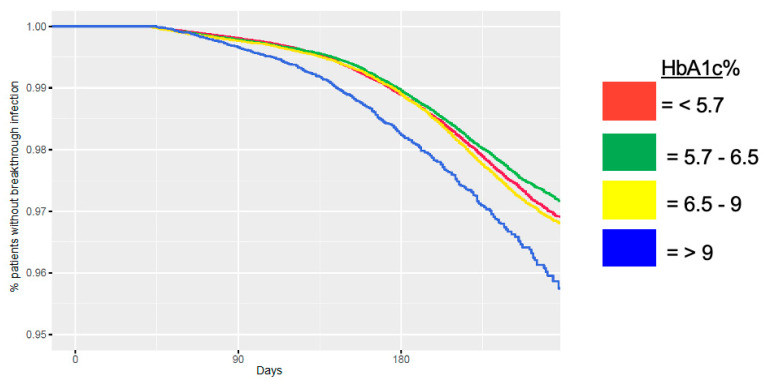
HbA1c and rate of breakthrough infection over time since second COVID-19 vaccination dose.

Using multivariable logistic regression, we then modeled the probability that someone would be hospitalized, with COVID-19 disease as the primary inpatient diagnosis, within thirty days of their breakthrough infection. Adjusting for the same variables as in the prior analysis, among patients with breakthrough COVID-19 infections, a one-point increase in BMI or HbA1c portends a statistically significant increase in the odds of hospitalization with a primary diagnosis of COVID-19. The odds ratio (OR) for risk of hospitalization from a breakthrough infection in the model with BMI, without HbA1c, was 1.017 (*p* = 0.001). The OR for the risk in the model with HbA1c, without BMI, is 1.158 (*p* < 0.001). When both BMI and HbA1c were added to the model, the HR were 1.010 (*p* = 0.053) and 1.063 (*p* < 0.001), respectively. In this case, a higher HbA1c again shows a higher risk of hospitalization than higher BMI (Table 5). Again, using a Kaplan–Meier curve, we show that patients with the highest HbA1c, which we delineated as >9%, have a disproportionately higher rate of breakthrough infection than patients with lower HbA1c (Figure 3).

## 4. Discussion

In summary, our results show that BMI and HbA1c are associated with increased risk of infection and hospitalization in breakthrough COVID-19 infections in our cohort of 9146 fully vaccinated veterans in the U.S. Veterans health system through October 2021. We show that there are some statistically significant, but not necessarily clinically significant, differences in mean BMI and HbA1c among the breakthrough and non-breakthrough infection groups. For example, the differential between a HbA1c of 6.2% in the non-breakthrough infection group is not necessarily clinically significant from an HbA1c of 6.34% in the breakthrough infection group. In clinical terms, this A1c difference would mean a 4 mg/dL difference in average blood sugar between groups. The reason for statistical significance is the large number of patients included in the analysis. Our analysis showed, using Cox proportional hazards ratio and multivariable logistic regression, that patients with higher BMI and HbA1c are at higher risk of breakthrough infection and of hospitalization due to a breakthrough infection of COVID-19.

Other recent studies have similarly investigated the role of metabolic parameters in the risk of breakthrough COVID-19 infections. A cross-sectional study of patients with obesity (BMI > 30 kg/m_2_) conducted using an online survey published in April 2022 found that a significantly higher prevalence of grade III obesity was detected in subjects with COVID-19 vaccine breakthrough infection compared to those without grade III obesity. The authors also found that there was a higher prevalence of type 2 diabetes and hypertension in respondents who had completed a third dose of a vaccine compared to those who had only completed one or two doses [14]. A study in Yale New Haven health system in 2021 found that among fully vaccinated patients who developed severe breakthrough infections, obesity and type 2 diabetes were among the pre-existing comorbidities in those patients [15]. In the UK, a COVID-19 symptom study of patients’ self-reported data found that individuals without obesity (BMI < 30 kg/m^2^) had lower odds of infection after their first vaccine dose than those with obesity [16]. Initial reports from the COVID-19 Corporate Data Warehouse included five VA centers reporting effectiveness of 75–86% after a second vaccine dose by antibody testing [17]. Our study adds specific metabolic comorbidities using clinical parameters, different from the diagnosis code inclusion by Bajema and colleagues [17]. A subsequent larger study of the full VA Corporate Data Warehouse reviewed 780,225 veteran records from March–December 2021, finding upwards of 84% effectiveness for the Pfizer vaccine in preventing breakthrough COVID-19 infection, decreasing by vaccine type and older age [18].

Given that both elevated BMI and diabetes are known risk factors for worse outcomes in COVID-19 infections, these results are not surprising. The proposed mechanisms for a risk of worse outcomes in COVID-19 infections prior to the advent of vaccinations, such as increased inflammation and impaired immune response, likely contribute to the association between higher BMI and A1c and risk of breakthrough COVID-19 infection seen in this analysis.

Our study population is predominantly made up of older white, non-Hispanic males, which is not reflective of the general U.S. population, but is representative of the national VA population. These findings may not generalize to patients outside the VA system. We do not capture breakthrough infections among veterans that occurred outside the VA system, which can be seen as a limitation; however, veterans tend to seek a majority of their care within the VA system. Another consideration is the fact that of those patients who were included in the analysis, those with available BMI and hemoglobin A1c data may have been more likely to have an incident positive COVID-19 test, since they are likely to have most of their care at the VA. Similarly, veterans with disabilities are included in this analysis, which limits our results by possibly skewing BMI and comorbid COVID-19 infections. The COVID-19 infections captured during this analysis are limited to those variants of the virus that were predominant during that time, and further analyses may benefit from capturing and perhaps stratifying by different variants of the virus. In addition, we controlled for important confounders such as sex, age, BMI, and A1c; however, important factors such as smoking status, medication use (particularly antidiabetics), comorbidities, and vaccination type and number of doses remain unaccounted for, as a limitation. Of relevance is the fact that since 2021, metformin and sitagliptin/spironolactone have been prospectively studied to mitigate COVID-19 and its complications, with encouraging early results [19,20]. The end of our analysis time frame in October 2021 limits the overlap with the rollout of the COVID-19 booster vaccination in subsequent months. Our study demonstrates the need for ongoing interventions to address obesity and glycemic control among our patients, both on an individual level and on a public health level. Perhaps better weight and glycemic control can ameliorate the risk of breakthrough infection or hospitalization due to COVID-19 infection.

## Figures and Tables

**Table 1:** Patient characteristics.

Characteristic	Category	*N* (% of Total)		No Breakthrough Infection *N* (% in Category)		Breakthrough Infection *N* (% in Category)	
Total	Vaccinated patients	549,344 (100.0%)		440,868 (80.2%)		4090 (0.75%)	
Race	White	357,929 (65.2%)		292,544 (66.4%)		2628 (64.2%)	
	Black or African American	123,230 (22.3%)		94,261 (21.4%)		951 (23.7%)	
	Multi-race	22,012 (4.0%)		17,245 (3.9%)		167 (4.1%)	
	Asian	6627 (1.2%)		5361 (1.2%)		429 (0.7%)	
	Other	8526 (1.6%)		6733 (1.2%)		60 (0.01%)	
	Missing	32,020 (5.8%)		24,724 (5.6%)		255 (6.2%)	
Ethnicity	Not Hispanic or Latino	437,453	(79.6%)	354,718	(80.4%)	3173	(77.6%)
	Multi-ethnic	47,764	(8.7%)	36,506	(8.3%)	394	(9.6%)
	Hispanic or Latino	42,646	(7.8%)	33,516	(7.6%)	362	(8.9%)
	Missing	21,481	(3.9%)	16,128	(3.7%)	161	(3.9%)
Sex	M	490,892	(89.4%)	397,764	(90.2%)	3665	(89.6%)
	F	58,452	(10.6%)	43,104	(9.8%)	425	(10.4%)
Age (years)	Median	67		69		65	
	Mean (SD)	64.5	(14.3)	66.1	(13.3)	63.2	(13.9)

**Table 2:** BMI among vaccinated patients.

Variable	Category	*N*	Value	% of Total	No Breakthrough Infection *N* (% in Category)		Breakthrough Infection *N* (% in Category)	
Body Mass Index (BMI)	Missing data	33,698		6.1%	26,066	(5.9%)	520	(8.2%)
	Available data	518,958		93.9%	414,868	(94.1%)	5802	(91.8%)
	Median		29.6		29.7		30.5	
	Mean (SD)		30.3	(6.1)	30.4	(6.0)	31.2 *	(6.2)

* *p* < 0.001 versus no breakthrough infection.

**Table 3:** Hemoglobin A1c among vaccinated patients.

Variable	Class	Variable	*N*% of Total		Value	No Breakthrough Infection *N* (% in Category)		Breakthrough Infection *N* (% in Category)	
HbA1c (%)	Missing		45,127	(8.2%)		33,981	(7.7%)	380	(9.3%)
	Available data		504,217	(91.8%)		406,887	(92.3%)	3710	(90.7%)
		Median			5.8	5.8		5.9	
		Mean (SD)			6.18 (1.27)	6.20	(1.24)	6.34 *	(1.5)

* *p* < 0.001 versus no breakthrough infection.

**Table 4:** Cox proportional hazards regression; HbA1c is a greater hazard for breakthrough infection than BMI.

	Model Without BMI or HbA1c			Model with BMI			Model with HbA1c			Model with BMI and HbA1c		
	Coefficient	HR Change per Unit Change	*p*-Value	Coefficient	HR Change per Unit Change	*p*-Value	Coefficient	HR Change per Unit Change	*p*-Value	Coefficient	HR Change per Unit Change	*p*-Value
Female	−0.182	0.834	3.49 × 10^−6^	−0.183	0.833	3.04 × 10^−6^	−0.165	0.848	0.000	−0.168	0.845	1.72 × 10^−1^
Age	−0.007	9.93 × 10^−1^	3.70 × 10^−15^	−0.006	9.95 × 10^−1^	3.55 × 10^−10^	−0.008	9.92 × 10^−1^	6.06 × 10^−19^	−0.007	9.94 × 10^−1^	2.78 × 10^−13^
BMI				0.017	1.017	1.16 × 10^−23^				0.01	1.015	1.89 × 10^−16^
HbA1c							0.07	1.077	6.07 × 10^−22^	0.06	1.063	2.83 × 10^−14^

**Table 5:** Logistic regression; both BMI and HbA1c are risk factors for hospitalization from breakthrough infections.

	Model Without BMI or A1c			Model with BMI			Model with A1c			Model with BMI and A1c		
	Coefficient	OR Change per Unit Change	*p*-Value	Coefficient	OR Change per Unit Change	*p*-Value	Coefficient	OR Change per Unit Change	*p*-Value	Coefficient	OR Change per Unit Change	*p*-Value
Female	−0.450	0.637	4.84 × 10^−3^	−0.457	0.633	4.28 × 10^−3^	−0.402	0.669	1.22 × 10^−2^	−0.408	0.665	1.10 × 10^−2^
Age	0.060	1.062	4.42 × 10^−87^	0.062	1.064	2.22 × 10^−87^	0.060	1.062	1.15 × 10^−85^	0.062	1.064	3.08 × 10^−84^
BMI				0.017	1.017	0.001				0.010	1.010	0.053
HbA1c							0.1468	1.1581	1.37 × 10^−13^	0.1393	1.1495	6.47 × 10^−12^

## Data Availability

The VA system does not make data publicly available.

## Abbreviations

The following abbreviations are used in this manuscript:

BMI	Body Mass Index
COVID-19	SARS Coronavirus-19
HbA1c	Hemoglobin A1c
HR	Hazard Ratio
OR	Odds Ratio
US	United States
VA	Veterans Affairs
VINCI	VA Informatics and Computation Infrastructure
